# Successful Endovascular Treatment of Delayed Spontaneous Intracranial Internal Carotid Artery Blowout Following Orbital Exenteration and Radiation

**DOI:** 10.7759/cureus.11535

**Published:** 2020-11-17

**Authors:** Laura Reed, Kristopher Lyon, Ethan A Benardete

**Affiliations:** 1 Neurosurgery, Baylor, Scott & White Healthcare, Temple, USA

**Keywords:** lacrimal carcinoma, cavernous carotid, carotid blowout, endovascular, orbital exenteration

## Abstract

Most head and neck cancers require aggressive surgical resection followed by external beam radiation therapy. The carotid artery can be injured by surgery or radiation resulting in a delayed “blowout.”

A patient who had undergone orbital exenteration for a lacrimal adenoid cystic carcinoma (ACC) followed by external beam radiation presented 16 years later with arterial bleeding from the orbit caused by “blowout” of the cavernous internal carotid artery (ICA). We review the literature on carotid blowout syndrome (CBS) and treatment.

The patient was emergently transferred to a hybrid operating room and underwent a balloon occlusion test (BOT) and endovascular sacrifice of the ICA with no neurological deficits postoperatively.

Emergent endovascular embolization is an effective treatment for an intracranial ICA blowout in this first reported case of a blowout through the orbit. Elevated radiation dose and lack of tissue coverage may put the cavernous ICA at risk for this delayed complication.

## Introduction

Aggressive surgical resection plus external beam radiation therapy (XRT) is the standard of care for most head and neck cancers. For cancers involving the orbit, perineural or bony invasion are indications for exenteration. After extensive resections, the carotid can become exposed or the wall weakened increasing the risk for rupture, which usually occurs within three months after surgery [1-2]. The patient in this report underwent orbital exenteration for lacrimal adenoid cystic carcinoma (ACC) without free flap coverage of the exposed orbit and dura at another institution as well as XRT. Sixteen years later, he experienced a carotid blowout syndrome (CBS) causing life-threatening hemorrhage from the orbit. The patient was successfully treated with emergent endovascular carotid sacrifice after passing a balloon occlusion test (BOT). This is the first reported case in the literature of intracranial CBS.

## Case presentation

The patient is a 72-year-old male with a history of ACC of the right nasal lacrimal duct treated with orbital exenteration followed by adjuvant radiotherapy of 54 Gy in 27 fractions followed by a stereotactic radiosurgery boost to the right supraorbital dura of 13 Gy 16 years prior to this presentation. Five years after his initial surgery, he had recurrence of the ACC to a right neck level II lymph node, and he subsequently underwent radical resection with adjuvant radiotherapy. His oncologic history is also significant for B cell lymphoma and prostate cancer, which were treated with chemotherapy and radiation, respectively and he was maintained on prophylactic warfarin because of a mechanical heart valve.

**Figure 1:**
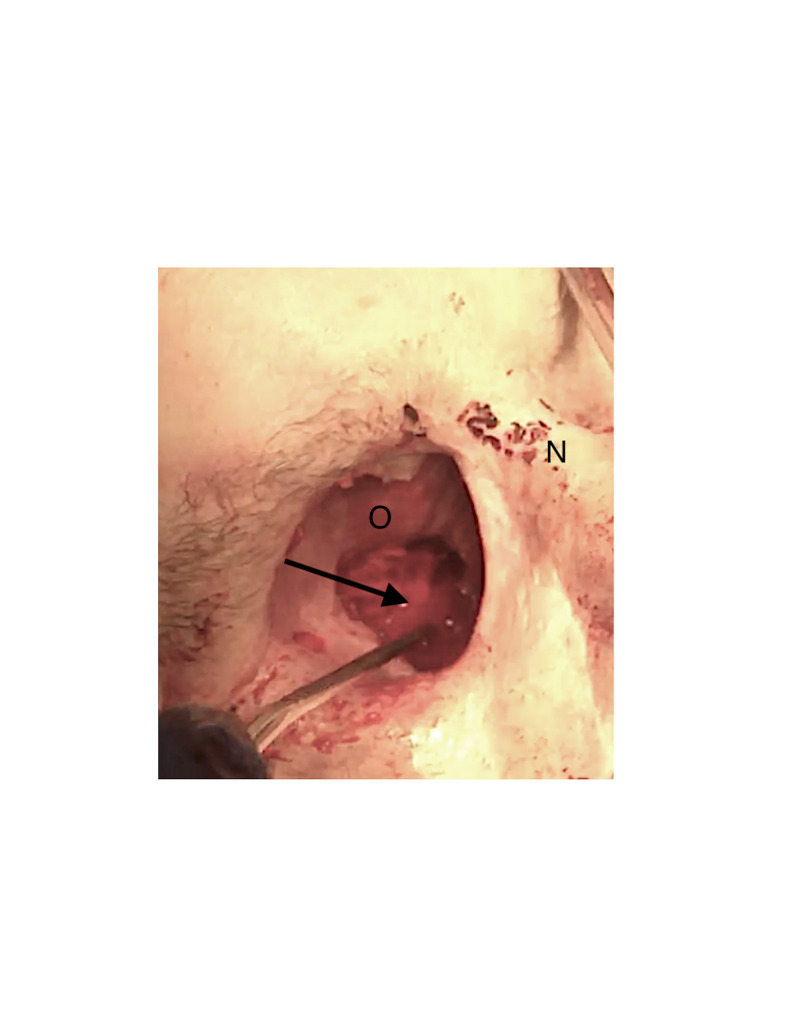
Active orbital bleeding. Smartphone picture of the orbit with active hemorrhage designated by the tip of the suction. The arrow indicates the point of bleeding.  O indicates the exenterated orbit.  N indicates the nasal bridge.

The patient presented to the ED with uncontrollable bleeding from his right orbit. An estimate of blood loss based on a comparison of the patient’s current hemoglobin to his baseline was three units. Warfarin was reversed, and the patient began receiving a transfusion of packed red blood cells. On examination, the orbital defect exposed a small area of dura and the right nasal cavity. A CT scan was obtained that showed no intracranial bleeding (not shown). Temporary hemostasis was obtained in the ED with a plug of bone wax (Figure 1). He was emergently transferred to the hybrid operating room (OR) suite.

Intervention

**Figure 2:**
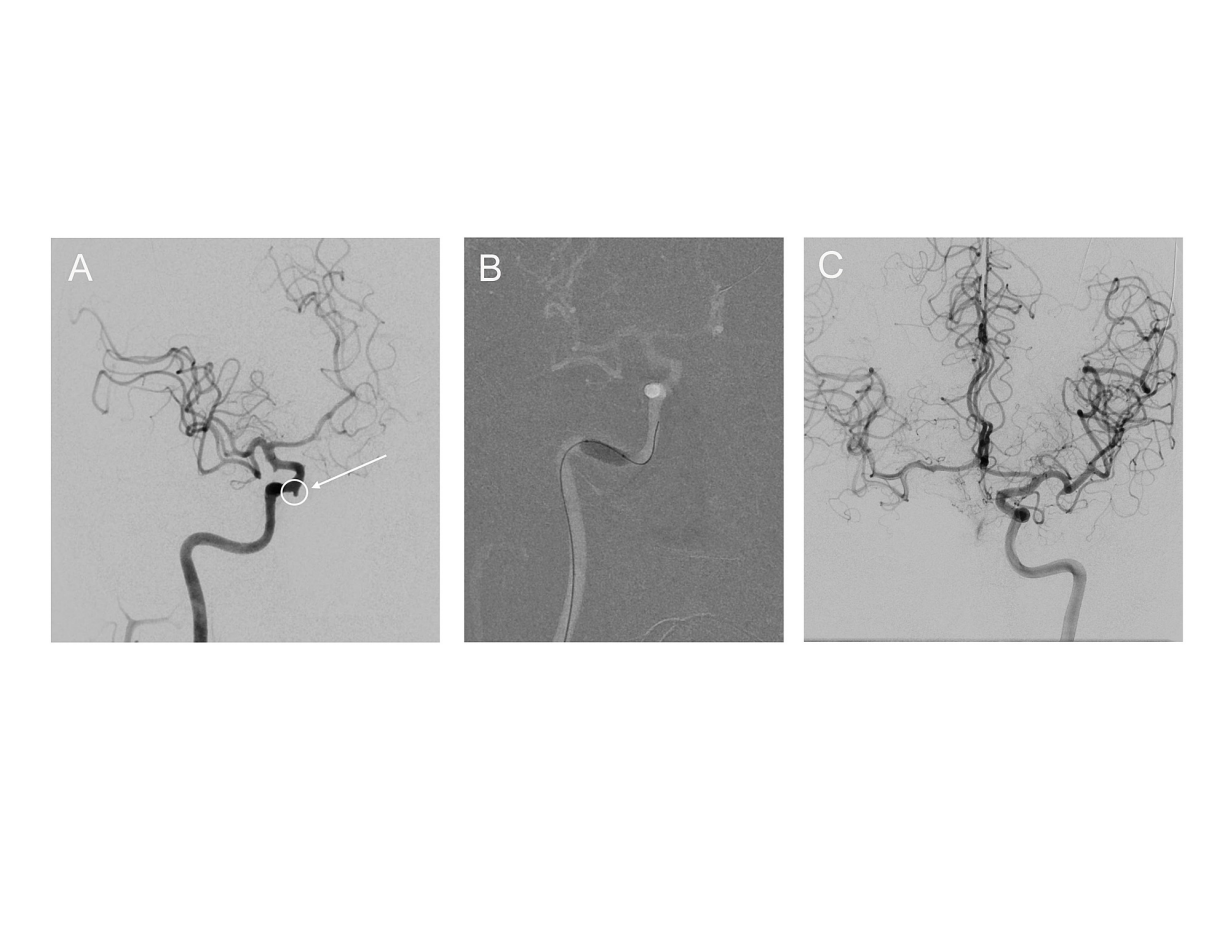
Endovascular evaluation of the hemorrhage source. Endovascular evaluation of the hemorrhage source.  A) Right internal carotid angiogram showing a cavernous pseudoaneurysm (white arrow, circle). B) Balloon occlusion of the right ICA. C) Excellent collateral filling of the right anterior circulation from the left sided ICA injection with the balloon inflated. ICA, internal carotid artery

The patient was intubated and general anesthesia was induced upon arrival. Somatosensory evoked potentials (SSEP) and electroencephalography (EEG) were monitored. Transfemoral digital subtraction angiography (DSA) of the right internal carotid artery (ICA) demonstrated a 3 mm bleb on the medial surface of the cavernous right ICA representing a pseudoaneurysm (Figure 2A). A second 5 Fr diagnostic catheter was placed in the left ICA via a left transfemoral approach. A 5000-unit bolus of IV heparin was administered, and activated clotting time (ACT) values were kept greater than 250 s for planned BOT and coil embolization. A 7 mm x 10 mm balloon (Transform, Stryker Neurovascular, Inc., CA, USA) was advanced into the horizontal petrous portion of the right ICA (Figure 2B). A BOT was performed with additional hypotensive challenge (20% below normal mean arterial pressure for 10 minutes) during which SSEP and EEG remained unchanged. Excellent cross filling across the anterior communicating artery was noted (Figure 2C). 

**Figure 3:**
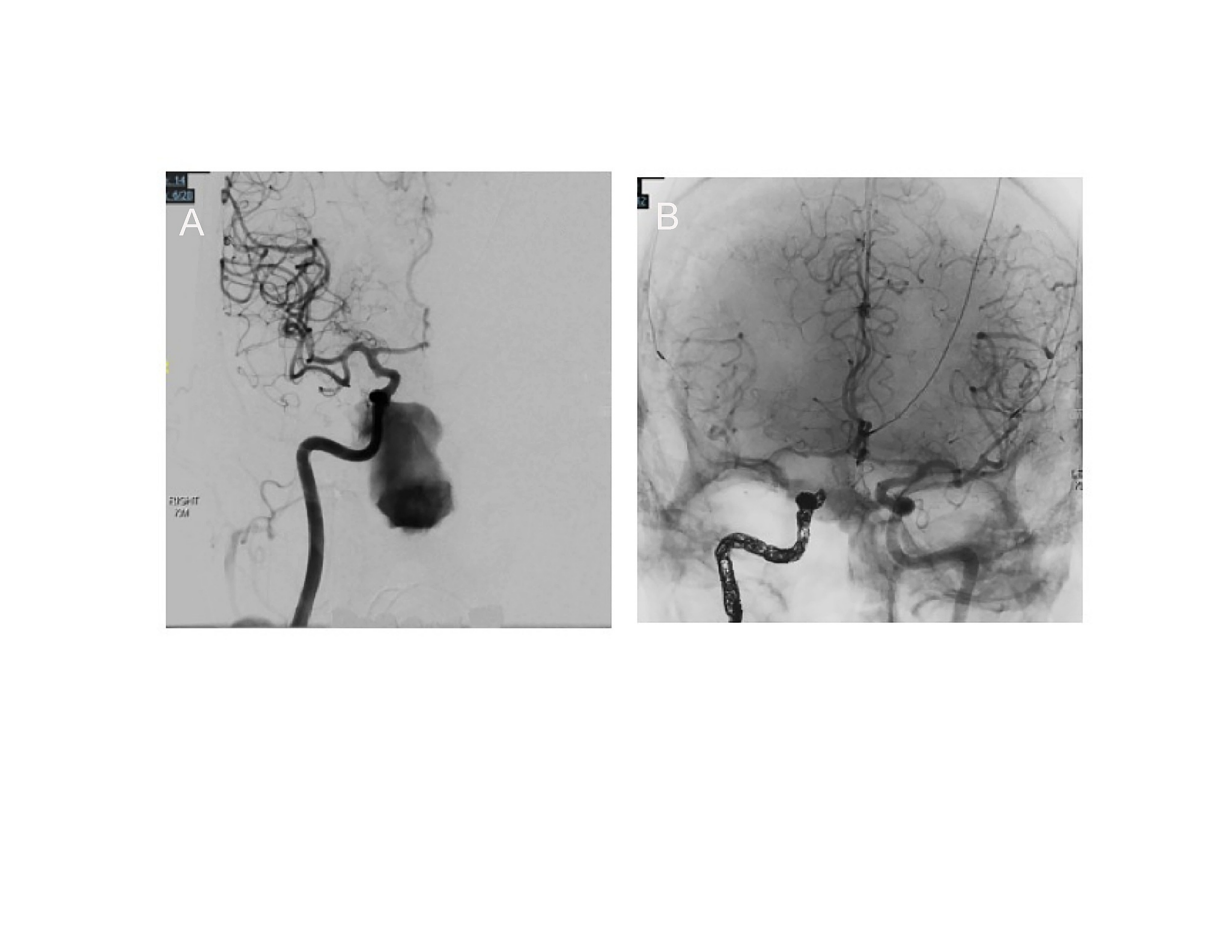
Intraoperative evaluation and repair of rupture. A) Active extravasation from the R ICA after removal of packing.  B) Left ICA angiogram after endovascular coil embolization of the R ICA with collateral filling of the right anterior circulation. The arrow indicates the coil pack.

The bone wax was removed from the orbit and angiography of the right ICA was performed, which demonstrated active extravasation from the pseudoaneurysm (Figure 3A). The site was packed again, and the decision was made to sacrifice the right ICA with coils (Target coils, Stryker Neurovascular, Inc., CA, USA) (Figure 3B). SSEP and EEG remained stable and postoperative angiography demonstrated excellent collateral filling of the right anterior circulation. 

Postoperatively, the patient was extubated in the ICU and had no further bleeding. He developed no neurological deficits and resumed anticoagulation. At one month and one year postoperatively, he had no further arterial bleeding. No evidence of recurrence of ACC was found by positron emission tomography; however, about nine months after intervention, he developed cerebrospinal fluid (CSF) leakage from the periorbital dura and underwent surgical repair. Over a year after treatment, the patient entered hospice care while undergoing treatment for recurrent extracranial lymphoma and expired.

## Discussion

Surgery and XRT can damage the carotid arteries leading to delayed spontaneous rupture or blowout [1]. Standard treatment for head and neck cancers with extension into the orbit, skull base, and nasal cavity consists of aggressive surgical resection followed by adjuvant radiation therapy. ACC of the lacrimal gland requires aggressive local treatment often including orbital exenteration followed by radiotherapy due to the high likelihood of perineural and bony invasion. Resection can damage or expose the carotid sheath or wall, as well as decrease blood flow to the adventitia of the artery leading to increased risk of rupture [2]. When a large vessel rupture does occur, it is generally happens from 10 days to three months after surgery; however, pseudoaneurysms after radical neck dissections have been found after 2-20 years [3-4]. Although endovascular management has been successfully used to treat CBS in multiple series, these cases involved the external carotid arteries, common carotid arteries, or extracranial internal carotid arteries. This is the first reported case of a spontaneous cavernous segment ICA blowout with successful endovascular treatment.

Carotid blowout syndrome can occur in 3%-4.5% of those radiated for head and neck cancers and in 4.5%-21% of those with repeat radiation [4]. The options for treatment of external, internal, or common carotid artery rupture are open repair, ligation, endovascular embolization, or stent grafting. Open repair is rarely chosen due to the high morbidity and mortality but can be helpful for source control before endovascular management. The mortality of those who required ligation of the extracranial CCA or ICA ranged from 15% to 100% with 10%-20% of the survivors having neurologic deficits [4]. Endovascular intervention, specifically embolization, has a lower morbidity and mortality when utilized properly and can decrease rebleeding events [4]. However, 8%-14% of patients can incur a permanent ischemic insult from embolization of the affected artery [4]. In 2018, a study by Wong et al. found that out of 266 patients, there was a 10.3% complication rate of ischemic events after embolization of the CCA or ICA and a 2.5% complication rate after stent reconstruction [5]. Rebleeding occurred in 31.9% of those treated with stents and in 9.1% of those with therapeutic endovascular occlusion. A BOT to evaluate the patient’s tolerance to a potential occlusion of the artery is recommended when feasible. However, as demonstrated in a large meta-analysis of 559 patients by Bond et al., up to 20% of the patients who passed a BOT had a delayed ischemic event [6]. Endovascular reconstruction of the damaged artery with stents to decrease the risk of neurologic sequela in those who fail a BOT may be feasible, but can result in higher rates of rebleeding and delayed stroke [5, 7].

A significant risk factor for CBS is a high total dose of radiation. The patient in this report had 67 Gy of radiation to the orbit and dura. In addition, the lack of tissue coverage of his orbital defect after exenteration left his cavernous sinus and frontal dura exposed. It is commonly recommended that a vascularized flap or skin grafts be performed for all patients to protect the intracranial elements after exenteration [8]. Other risk factors for CBS include recurrence of tumor, poor nutrition, diabetes, and steroid use [9]. Active extravasation from the ruptured artery is categorized as the most severe, highest mortality grade, namely 4 [10]. After passing the BOT, coil embolization of the damaged intracranial ICA was performed without rebleeding or neurological deficit. 

## Conclusions

Embolization is a safe and effective way to treat an intracranial ICA blowout, and this is the only documented case of a successful endovascular embolization of an intracranial ICA blowout without neurologic sequelae. Although prior studies discuss extracranial CBS following head and neck cancer surgery with or without adjuvant radiation, this patient may be one of the few survivors of an intracranial carotid artery blowout, thus limiting the ability to compare the treatment modalities for this complication. A comprehensive registry of patients who have suffered intracranial ICA blowout following head and neck surgery would shed light on the actual variety of treatments and outcomes.
